# Diagnostic re-classification and prognostic risk stratification of patients with acute chest pain

**DOI:** 10.1007/s12471-019-01328-6

**Published:** 2019-08-29

**Authors:** J. W. Deckers

**Affiliations:** Department of Epidemiology, Erasmus MC, Rotterdam, The Netherlands

**Keywords:** Acute coronary syndromes, Myocardial infarction, Unstable angina, Diagnosis, Prognosis

## Abstract

Unstable angina and myocardial infarction are prevalent manifestations of acute coronary artery disease, combined in the term ‘acute coronary syndromes’. The introduction of sensitive markers for myocardial necrosis has led to confusion regarding the distinction between small myocardial infarctions and ‘true’ unstable angina, and the application of ever more sensitive markers has accelerated the pace at which patients with unstable angina are being re-classified to non-ST-segment elevation myocardial infarction. But in how many patients with acute chest pain is myocardial ischaemia really the cause of their symptoms? Numerous studies have shown that most have <5 ng/l high-sensitivity cardiac troponin, and that their prognosis is excellent (event rate <0.5% per year), incompatible with ‘impending infarction’. This marginalisation of patients with unstable angina pectoris should lead to the demise of this diagnosis. Without unstable angina, the usefulness of the term acute coronary syndromes may be questioned next. It is better to abandon the term altogether and revert to the original diagnosis of thrombus-related acute coronary artery disease, myocardial infarction. A national register should be the next logical step to monitor and guide the application of effective therapeutic measures and clinical outcomes in patients with myocardial infarction.

The best-known and most prevalent manifestations of acute coronary artery disease include unstable angina and myocardial infarction (MI), the latter comprising both fatal and non-fatal events. For a long time, these diagnoses have been combined in the term ‘acute coronary syndromes’ (ACS). One of the earliest descriptions of this syndrome was published in 1988 [1]. In fact, the pathophysiology of the two types of MI, nowadays known as ST-segment elevation and non-ST-segment elevation MI, has not changed significantly since Fuster’s comprehensive review on ACS 30 years ago. That, however, is not the case for unstable angina.

## The ‘creation’ of unstable angina

For a considerable time, unstable angina has been considered to be a heterogeneous syndrome [2]. In the past, verbatim terms were employed to describe its most likely and often ominous clinical course (Tab. 1). The categorisation of the various clinical entities by Braunwald into one diagnostic category, unstable angina pectoris, was therefore timely. He classified unstable angina into three clinical circumstances in which angina occurred: (1) secondary (e.g. in the presence of severe anaemia), (2) post-infarction angina, when angina pectoris developed immediately after MI, and (3) otherwise as primary unstable angina. The diagnosis was further stratified according to its severity. In total, nine categories of unstable angina pectoris could be distinguished although, based upon the intensity of its treatment and the presence or absence of transient ECG abnormalities, further sub-classification was possible. A tenth category of unstable angina was added later following the recognition that elevated levels of cardiac markers for myocardial damage were strongly associated with adverse outcomes in patients with acute chest pain [3]. While this adapted classification rightfully acknowledged the diagnostic and prognostic importance of cardiac biomarkers, the new definition led to confusion regarding the distinction between ‘small’ MIs and ‘true’ unstable angina, chest pain resulting from myocardial ischaemia in the absence of myocardial injury.

**Table 1:** The classification of angina pectoris at rest in different eras [2, 6, 7]

Early	Late	Present
Crescendo angina Accelerated angina Pre-infarction angina Impending infarction Post-infarction angina Intermediate coronary syndrome Acute coronary insufficiency Status anginosus	Unstable angina pectoris: – Primary – Secondary – Post-infarct Subcategories: – Severity of symptoms – ECG changes	Angina pectoris class IV CCS

*CCS* Canadian Cardiovascular Society

## Its disappearance

**Fig. 1:**
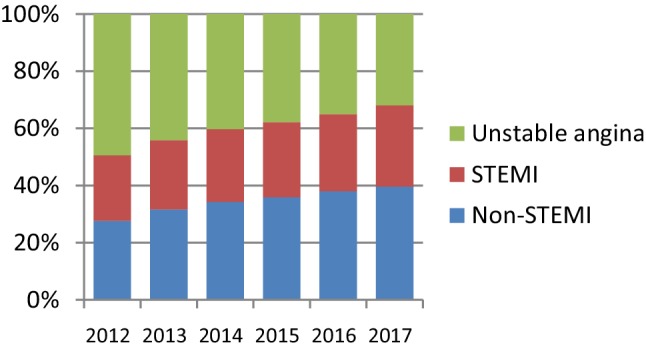
Proportion of Dutch patients categorised as non-ST-segment elevation myocardial infarction (*Non-STEMI*), ST-segment elevation MI (*STEMI*) and unstable angina pectoris in the last 6 years. Based upon ‘diagnosis-related groups’. (Source: Nederlandse Zorgautoriteit http://www.opendisdata.nl)

The introduction of newer and more sensitive markers for myocardial necrosis from 2010 onwards intensified this debate. While the overall number of patients with MI decreased, a shift was observed from the diagnosis of unstable angina to non-ST-segment elevation MI, resulting in an increase in the number of patients with a small MI [4, 5]. The clinical application of even more sensitive cardiac biomarkers, including the high-sensitivity cardiac troponin assays, accelerated the pace at which patients with unstable angina were being re-classified to non-ST-segment elevation MI. Dutch figures on the number of patients with unstable angina reflect this phenomenon. In 2013, no less than about 50%—almost 30,000—of all patients hospitalised with ACS were diagnosed as unstable angina pectoris. Since then, the number of Dutch patients with a non-ST-segment elevation MI has gradually increased, to the further detriment of the number of patients with unstable angina. Their total dropped to 20,000 in the year 2016 (Fig. 1). Since then, the proportion of ACS patients with unstable angina has declined further, although no absolute figures for the most recent years are available. This so-called ‘marginalisation’ of patients with unstable angina pectoris did not escape Braunwald, and resulted in his ‘requiem’ for unstable angina in 2013 [6]. From then on, patients with angina at rest were to be classified as ‘angina pectoris class IV’ according to the customary and well-known Canadian Cardiovascular Society grading of angina pectoris [6, 7].

## And its demise

Nevertheless, the answer to the question as to how many patients with acute chest pain truly have coronary insufficiency—myocardial ischaemia—as the cause for their symptoms remains elusive. As foreseen, ever more sensitive markers for myocardial damage have been introduced and clinically applied in large numbers in recent years. In fairness, it is unclear whether their application has had an effect on the overall prognosis of patients with acute chest pain [8]. But it has become very clear that the prognostic stratification of patients with acute chest pain has benefitted greatly from the use of these new assays. Numerous studies have now shown that the prognosis of patients with very low or non-detectable levels of high-sensitivity cardiac troponin assays is excellent. The Scottish study of Shah and co-workers provides a fine example thereof [9]. In that study of 6.304 consecutively observed patients with acute chest pain, 16% were diagnosed with MI. Among the other 84%, the large majority had high-sensitivity cardiac troponin levels <5 ng/l. The prognosis of these patients was excellent, with less than 0.5% of them experiencing a cardiovascular event in the next 500 days. That is comparable to the risk of asymptomatic men and women of about 60 years of age. Additional diagnostic and prognostic stratification in such patients is usually unnecessary. The other, relatively small, group of patients with a slightly higher troponin concentration—albeit within the (normal) 99th percentile range—was at somewhat higher risk, although probably not high enough to justify elaborate diagnostic or therapeutic interventions other than (secondary) preventive measures when and where indicated.

## Next: from ACS to MI

Without the (now useless) diagnosis of unstable angina pectoris, the time has come to question the usefulness of the term acute coronary syndromes since, from now on, this entity comprises only ST-segment elevation and non-ST-segment elevation MI. Moreover, one must consider the fact that some of our colleagues exploit the term ACS in order to add other diagnoses to that category. For instance, a recent editorial suggested including ‘Takotsubo cardiomyopathy’ within ACS, just because its early clinical presentation may mimic the presence of ST-segment elevation MI [10]. The pathophysiology of the cardiomyopathy, however, could not be more different than that of acute MI. Other diseases with infarct-like early clinical presentations, such as acute pericarditis or aneurysm of the ascending thoracic aorta that occludes the origin of one of the coronary arteries, can also resemble ST-segment elevation MI, but certainly should not become part of ‘ACS’ for the same reason. The bottom line is: when the pathophysiology of a clinical entity has been uncovered, the word ‘syndrome’ should no longer be employed.

**Fig. 2:**
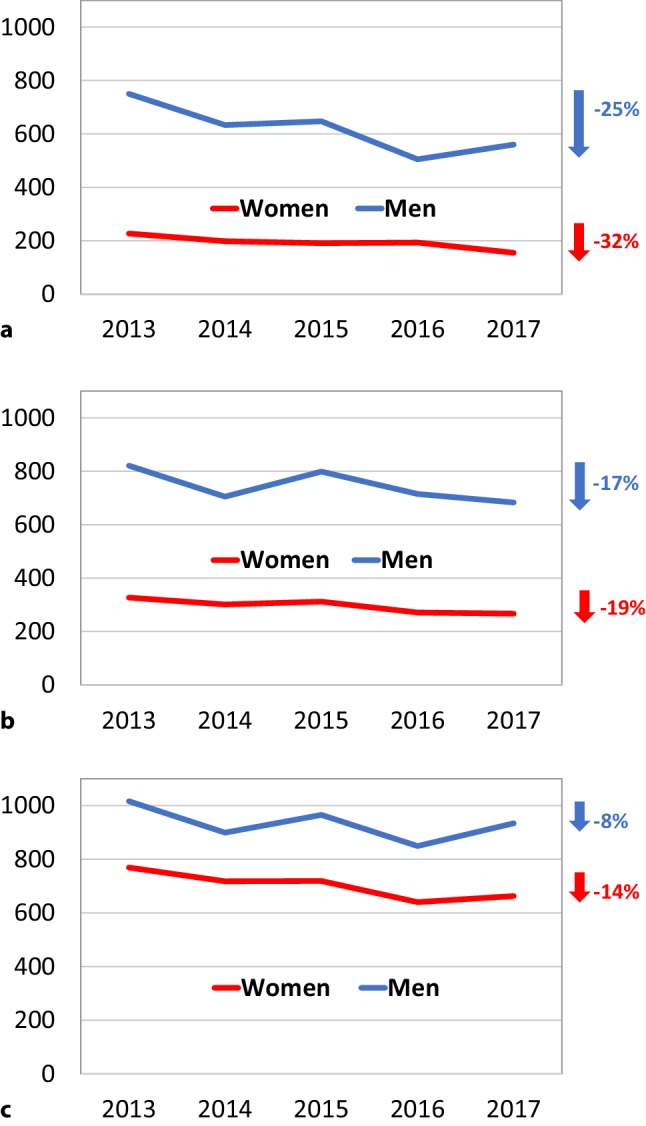
Mortality (in and outside hospital) from myocardial infarction in different age groups, in women and men, in the last 5 years. Crude numbers (standardisation unnecessary given limited time frame) and relative changes (in percentages) between the years 2013 and 2017. **a** Women and men <65 years of age. **b** Women and men between 65 and 75 years of age. **c** Women and men between 75 and 85 years of age. (Source: Voor wat er feitelijk gebeurt. CBS Dataportaal ©CBS, 2018)

Thus, it may be better to abandon the term ACS altogether and go back to the original diagnosis of thrombus-associated acute coronary disease, myocardial infarction. Other reasons for doing so include the large number of subjects affected and, despite the availability of effective therapeutic options, the high event rate that goes with the diagnosis. Fortunately, MI-associated mortality is still decreasing, both in Dutch women and men (Fig. 2). Measures to further reduce MI-related case fatality, within and outside the hospital, will be extremely cost-effective. A national register would be a logical and valuable next step to monitor and guide the application of effective therapeutic measures and clinical outcomes of patients with MI.
